# Alumni Association of the Albany Medical College

**Published:** 1881-08

**Authors:** 


### Alumni Association of the Albany Medical College.

We would earnestly call the attention of every Alumnus of Albany Medical College to the following announcement:

At the meeting of the executive committee of the Alumni, held June 16, 1881, the president stated that he had given considerable thought to the matter of prize essays to increase the interest in and efficiency of the Association.

He had concluded to offer an annual prize of $100, to be called "A Surgical Prize," and would announce as the subject for this year, "An Essay on Colles Fractures, its Pathology and Treatment," to be accompanied with a pathological specimen illustrating the fracture, with or without dislocation of the ulua, or a careful dissection of the hand, wrist and fore-arm.

After favorable remarks by members of the committee, on motion of Dr. L. Hale, Dr. Van Deveer, the president, was appointed a committee of one to give the matter further consideration. Subsequently he reported that the heirs of the late Prof. Alden Marsh, M. D., L.L. D., desired to give the sum of $100 as an annual "Marsh Memorial Prize," the essay for the coming year to be on the "Pathology and Treatment of Morbus Coxarius."

Also, that Mr. A. McClure, a governor of the Albany Hospital, had decided to give the sum of $100 annually as an "Armsby Memorial Prize," the essay to be on some anatomical subject. That for the coming year will consist of a minute description of the genitc-urinary organs of the male, together with a carefully dissected specimen of the same.

The president further reported that the heirs of the late Prof. James MacNaughton, M. D., had offered the sum of $100 as a "MacNaughton Memorial Prize." The subject of the essay for the current year to be "Antisepsis in the Treatment of Diseases," and Mr. Joseph Russell, a trustee of the College, offers for this year a second surgical prize of $100 for the second best essay on "Colles Fractures."

Essays and specimens designated by a motto and accompanied by a sealed envelope inclosed with the same motto, and containing the name and address of the author, must be sent to the secretary, Dr. W. G. Tucker, by the 14th day of February, 1882.

The committee to examine the essays for this year will consist of Drs. A. Van Deveer, J. Murher and Lorenzo Hale, and they reserve the right to reject any or all the essays if not deemed worthy.

All specimens are to be deposited in the new museum of the College, properly labeled.

